# Yi Mai Granule Improves High-Fat Diet–Induced Nonalcoholic Fatty Liver Disease in Mice by Regulating Gut Microbiota and Metabolites

**DOI:** 10.1155/ijm/2273986

**Published:** 2025-03-23

**Authors:** Linlin Pang, Yongming Liu, Changbin Yuan, Yetao Ju, Junpeng Wu, Meijia Cheng, Sian Jin, Ying Fan, Huiyong Zhang, Yu Wang, Dongyu Min

**Affiliations:** ^1^Key Laboratory of Ministry of Education for Traditional Chinese Medicine Viscera-State Theory and Applications, Liaoning University of Traditional Chinese Medicine, Shenyang, Liaoning, China; ^2^Department of Cardiovascular Medicine, Affiliated Hospital of Liaoning University of Traditional Chinese Medicine, Shenyang, Liaoning, China; ^3^Experimental Center of Traditional Chinese Medicine, Affiliated Hospital of Liaoning University of Traditional Chinese Medicine, Shenyang, Liaoning, China; ^4^First Clinical College, Liaoning University of Traditional Chinese Medicine, Shenyang, Liaoning, China; ^5^College of Traditional Chinese Medicine, Liaoning University of Traditional Chinese Medicine, Shenyang, Liaoning, China; ^6^Department of Traditional Chinese Medicine, Fourth Affiliated Hospital of China Medical University, Shenyang, Liaoning, China

**Keywords:** gut microbiota, high-fat diet, metabolome, nonalcoholic fatty liver disease, traditional Chinese medicine

## Abstract

Yi Mai granule (YMG) is a traditional Chinese medicine (TCM) herbal decoction consisting of two TCM formulas: Gua-Lou-Ban-Xia decoction and Si-Jun-Zi decoction. YMG has shown clinical benefit in the treatment of nonalcoholic fatty liver disease (NAFLD), which may be due to its regulatory effects on lipid metabolism. Previous studies have highlighted the importance of the gut microbiota and its metabolites in the use of TCM. However, the effect of YMG on the gut microbiota in the treatment of NAFLD remains unclear. In this study, we established an NAFLD model in *ApoE^−/−^* mice and treated them with YMG. High-performance liquid chromatography was adopted to identify the chemical components of YMG. By mapping the candidate targets using network pharmacology, we found that the targets of the main components of YMG were significantly enriched in NAFLD-related pathways. Moreover, *16S rRNA* gene sequencing revealed that YMG affected the constitution and metabolism of the gut microbiota in NAFLD model mice, including lipid and carbohydrate metabolism. Similarly, metabolites related to lipid and carbohydrate metabolism in mouse serum were significantly altered by YMG. The correlation heat map and network analyses showed that the gut microbiota and metabolites affected by YMG were closely related to the blood lipid content. Collectively, YMG may exert therapeutic effects by affecting the metabolism of gut microbiota, thus regulating lipid and carbohydrate metabolism. These findings offer novel insight into the pharmacological mechanism of YMG in the treatment of NAFLD and provide theoretical bases for its clinical applications.

## 1. Introduction

Nonalcoholic fatty liver disease (NAFLD) is a common chronic disease, and its incidence rate is increasing rapidly worldwide annually [1, 2]. NAFLD leads to numerous liver diseases, including isolated steatosis, nonalcoholic steatohepatitis (NASH), liver fibrosis, cirrhosis, and hepatocellular carcinoma [3–5]. Although many researchers have studied its pathology and therapeutic drugs, the treatment options for NAFLD are still lacking due to the complexity of the disease mechanism and the lack of efficacious therapeutic drugs [3]. Therefore, the development of therapeutic drugs for NAFLD is urgently needed.

Numerous medicinal plants and their active ingredients used in traditional Chinese medicine (TCM) have shown significant effects in combating obesity and treating NAFLD [6, 7]. Yi Mai granule (YMG) is a TCM that includes nine medicinal plants: *Codonopsis pilosula* (Franch.) Nannf., *Atractylodes macrocephala* Koidz., *Poria*, *Radix Glycyrrhizae*, *Atractylodes macrocephala*, *Semen Persicae*, *Carthamus tinctorius* L., *Pinelliae Rhizoma Praeparatum Cum Alumine*, and *Trichosanthis Fructus*. In previous studies, YMG may alleviate high-fat diet (HFD)–induced atherosclerosis by activating mitochondrial autophagy [8]. However, due to the complexity of TCM ingredients and the multitarget nature of active ingredients, it is still unclear whether YMG can improve HFD-induced NAFLD. Recently, network pharmacology has been applied to study modern Chinese medicine by integrating bioinformatics and systems medicine that precisely conforms to the overall multicomponent and multitarget connotation of Chinese medicine. Network pharmacology can potentially reveal the mechanism of TCM's action by discovering its active ingredients and biomarkers.

Numerous recent studies have shown that the gut microbiota and its metabolites may be potential therapeutic targets for some active ingredients in TCM [9–11]. A study by Bi et al. [11] showed that the improvement and therapeutic effects of ZiBuPiYin, a TCM, on diabetes-associated cognitive decline were dependent on the gut microbiota and its metabolites. A study demonstrated that Jiangzhi particles could significantly modulate the functional profile of the gut microbiota in NAFLD model mice, decreasing lipopolysaccharide biosynthesis and sulfur metabolic pathways to ameliorate symptoms in the mice [9]. Therefore, the interaction between TCM and the gut microbiota in improving a disease is a key research direction. Understanding the mechanism of YMG in improving NAFLD mediated by the gut microbiota and its metabolites might help in understanding the mechanism of drug action and clinical applications.

In this study, the potential targets of YMG in improving NAFLD were screened using network pharmacology. *16S rRNA* gene sequencing and ultrahigh-performance liquid chromatography–tandem mass spectrometry (UPLC-MS) technologies were used to identify the gut microbiota and blood metabolites in *ApoE*^−/−^ mice and C57BL/6J mice fed a HFD. A complex regulatory network among YMG, the gut microbiota, its metabolites, and NAFLD symptom phenotypes was constructed. This study is aimed at explaining the mechanism of the drug action of YMG in improving NAFLD, thereby providing a molecular theoretical basis for the clinical treatment of and drug selection for NAFLD.

## 2. Materials and Methods

### 2.1. Animals

A total of 20 male *ApoE*^−/−^ mice (8 weeks old, weighing 18–20 g) and 10 male C57BL/6J mice (8 weeks old, weighing 18–20 g) were purchased from BinYue Laboratory Supplies Company in Shenyang, China. The mice were raised in the Scientific Research Center of Liaoning University of Traditional Chinese Medicine (Liaoning, China). The mice were reared in a specific pathogen-free (SPF) environment (23°C, with 12-h/12-h light/dark cycles, 65% humidity, and free access to food and water). Each mouse was housed individually in a cage. The experimental procedures were approved by the Animal Ethics Committee of Liaoning University of Traditional Chinese Medicine (Animal Ethics No. 2022CS (DW) -016-01).

### 2.2. Experimental Design

After one week of adaptive feeding, 12 *ApoE*^−/−^ mice were randomly divided into model and YMG groups (*n* = 6 per group), and 6 C57BL/6J mice were used as the control group. The control group mice were fed maintenance feed containing 4% fat, 18% crude protein, 5% crude fiber, etc., daily with free access to food and water. The model and YMG group mice were fed HFD, containing 21% fat, 10% lard, 1% cholesterol, etc., daily with free access to food and water. After a fortnight of normal feeding, the YMG group was given YMG potion daily via gastric lavage. The drug dose was determined by the ratio of the human body surface area to the animal body surface area. The YMG group mice were given 19.50 g/kg/day of the crude drug. According to the *Pharmacology Methodology of TCM*, People's Health Publishing House, first edition, September 1993, the drug dosages were determined by the ratio of the human body surface area to the animal body surface area, as given in Equation (1).

(1) $$\begin{array}{c} \text{Total amount of oral medication in mice }\left(\text{g}/\text{kg}/\text{day}\right)=\text{rotal amount of oral medication in humans }\left(75\text{g}/70\text{ kg}/\text{day}\right)\times \text{ratio of body surface area between humans and mice }\left(0.0026\right)\times 50 \end{array}$$

The control and model group mice were given an equal volume of normal saline every day. After 12 weeks of administration, mice were euthanized by intraperitoneal injection of pentobarbital sodium (150 mg/kg), and samples of cecal contents were collected for subsequent experiments.

### 2.3. Preparation of YMG and Identification of Its Chemical Composition

All medicinal materials in YMG were purchased from Shenyang Jinzhu Pharmaceutical Co. Ltd. The names and dosages of these materials are listed in Table 1.

After accurately weighing all the medicinal materials, water (eight times the amount of materials) was added three times for 1 h each time, followed by merging the decoction and filtration. The filtrate was then concentrated.

The formulated YMG tonic mixture was identified using an Agilent 6530 QToF mass spectrometer and an Agilent 1260 HPLC liquid chromatography. The separation was performed on a ZORBAX SB-C18 column (4.6 × 50 mm, 1.8 *μ*m). The column temperature was 30°C, and the injection volume was 5 *μ*L. The mobile phase consisted of 0.1% formic acid aqueous solution (A)–acetonitrile (B) with gradient elution from 0 to 60 min and a flow rate of 0.5 mL/min. The mass spectrometry was monitored by an electrospray ion source (ESI). The other UPLC-MS conditions were as follows: gas temperature, 350°C; gas flow, 8 L/min; sheath gas temperature, 380°C; sheath gas flow, 11 L/min; and scan range, 100–1200 *m*/*z*.

### 2.4. Measurement of Blood Lipids in Mice

Blood samples were collected from mice after anesthesia. The serum was isolated by centrifuging the blood samples at 3500 rpm for 25 min. An automatic biochemical analyzer (Toshiba, Japan) was used to measure the serum contents, including triglycerides (TGs), total cholesterol (TC), low-density lipoprotein cholesterol (LDL), and high-density lipoprotein cholesterol (HDL).

### 2.5. Pathological Observation of Liver Tissue

The liver of each mouse was fixed in 4% paraformaldehyde (Solarbio, China), dehydrated in ethanol, and paraffinized. The paraffinized tissues were sectioned into 4-*μ*m-thick sections and stained with hematoxylin and eosin (H&E) (Beyotime, Shanghai, China). Liver tissue sections were also stained with Oil Red O to further confirm the presence of steatosis. Liver sections were viewed under a light microscope (Nikon, Japan) to evaluate the cell organization in mouse livers. ImageJ software was used to measure the relative lipid area of each group stained with Oil-Red O.

### 2.6. Pharmacological Analysis of the YMG Network

The chemical constituents of RMKL were searched using “*Codonopsis pilosula* (Franch.) Nannf.,” “*Atractylodes macrocephala* Koidz.,” “*Poria*,” “*Radix Glycyrrhizae*,*”* “*Atractylodes macrocephala*,” “*Semen Persicae*,” “*Carthamus tinctorius* L.,” “*Pinelliae Rhizoma Praeparatum Cum Alumine*,” and “ *Trichosanthis Fructus*” as keywords in the TCMSP database (https://www.tcmsp-e.com/). Effective compounds were screened based on ≥ 30% oral bioavailability (OB) and ≥ 0.18% drug-likeness (DL), and their prediction targets were obtained. The YMG targets were obtained by merging and deleting weight.

The keyword “dyslipidemias” was searched in GeneCards (https://www.genecards.org/), CTD (https://ctdbase.org/), and DisGeNET (https://www.disgenet.org/) databases. The target protein information was transformed into gene names and UniProt IDs in the UniProt (https://www.uniprot.org) database, and the disease target of dyslipidemia was obtained after deletion.

The acquired YMG target was intersected with the disease target, which was the potential target of YMG's action on dyslipidemia. The intersection target was imported into the STRING (https://www.string-db.org/) database, and a protein–protein interaction (PPI) network was obtained by selecting “*Home sapiens*” species and a score of ≥ 0.7. The PPI network was imported into Cytoscape V3.9.0 software, and the topology of the PPI network was analyzed using the CytoHubba plugin. The first 40 targets of the four common algorithms (MNC, Degree, EPC, and MCC) were selected, and the intersecting genes were identified to select the core genes.

The intersecting target genes were imported into the DAVID (https://david.abcc.ncifcrf.gov/) database, and gene ontology (GO) and Kyoto Encyclopedia of Genes and Genomes (KEGG) analyses were performed after selecting the species “*Home sapiens.*” The WeChat online drawing website (http://www.bioinformatics.com.cn/) was used for visualization.

### 2.7. Western Blotting

For protein extraction, the tissue samples dissected from the mouse liver were lysed with 1% phosphatase inhibitor and protease inhibitor. After an incubation of 30 min, the supernatant was centrifuged at 4°C and 12,000 rpm for 10 min. The extracted protein samples were quantified using a bicinchoninic acid assay protein assay kit (Beyotime, China). All the previous steps were executed on ice. The samples were denatured in a Laemmli sample buffer containing sodium dodecyl sulfate (SDS), which was mixed with tissue protein at a ratio of 1:4 and subsequently boiled in a 100°C water bath for 5 min. The samples containing 50 *μ*g protein were separated using SDS–polyacrylamide gel electrophoresis (PAGE) and transferred to polyvinylidene fluoride membranes. They were blocked with 5% skimmed milk powder for 1 h and incubated overnight with antibodies against the following proteins: *β*-actin (1:1000), caspase-3 (1:1000), CYCS (1:1000), interleukin (IL)1*β* (1:1000), IL6 (1:1000), PPAR (1:1000), RXRA (1:1000), tumor necrosis factor (TNF) (1:1000), nuclear factor (NF)-*κβ* 65 (1:1000), and phosphorylated (p)-NF-*κβ* 65 (1:1000). The membranes were washed three times in tris-buffered saline with Tween-20 (TBS-T) for 10 min, incubated with the respective horseradish peroxidase–conjugated secondary antibodies for 1 h, and subsequently washed using the same protocol. An electrochemiluminescence (ECL) solution was used to cover the strip, and a photoluminescence imaging system (Tanon 520) was used to acquire blot images. Finally, gray values were measured using ImageJ software.

### 2.8. *16S rRNA* Gene Sequencing of Mouse Gut Microbiota

According to the DNA extraction and quality control method of Zhan et al. [12],the OMEGA Soil DNA Kit (M5635-02) (Omega Bio Tek, Norcross, Georgia, United States) was used to extract the total genomic DNA of microorganisms from mouse fecal samples. The quantity and quality of the extracted DNA were measured using a NanoDrop NC2000 spectrophotometer (Thermo Fisher Scientific, Waltham, Massachusetts, United States) and agarose gel electrophoresis, respectively.

### 2.9. *16S rRNA* Gene Amplicon Sequencing and Bioinformatics Analysis

Polymerase chain reaction (PCR) amplification of the bacterial *16S rRNA* gene V3–V4 region was performed using the forward primer 338F (5⁣′-ACTCCTACGGGAGGCAGCA-3⁣′) and the reverse primer 806R (5⁣′-GGACTACHVGGGTWTCTAAT-3⁣′) [13]. Sample-specific 7-bp barcodes were integrated into the primers to facilitate multiplex sequencing. The PCR mixture consisted of 5 *μ*L of buffer (5X), 0.25 *μ*L of Fast Pfu DNA Polymerase (5 U/*μ*L), 2 *μ*L of (2.5 mM) dNTPs, 1 *μ*L (10 *μ*M) each of forward and reverse primers, 1 *μ*L of DNA template, and 14.75 *μ*L of ddH_2_O. The thermal cycling protocol included an initial denaturation at 98°C for 5 min, followed by 25 cycles of denaturation at 98°C for 30 s, annealing at 53°C for 30 s, and extension at 72°C for 45 s, with a final extension at 72°C for 5 min. The PCR products were purified using Vazyme VAHTSTM DNA Clean Beads (Vazyme, Nanjing, China) and quantified using the Quant-iT PicoGreen dsDNA Assay Kit (Invitrogen, Carlsbad, California, United States). After quantification, amplicons were pooled in equimolar amounts and subjected to paired-end 2 × 250 bp sequencing on the Illumina NovaSeq platform with the NovaSeq 6000 SP Reagent Kit (500 cycles) at Shanghai Personal Biotechnology Co. Ltd (Shanghai, China).

The analysis of gut microbiota was performed using QIIME2 2019.4 following the official tutorials (https://docs.qiime2.org/2019.4/tutorials/) with slight modifications [14]. Briefly, raw sequence data were demultiplexed using the demux plugin, followed by primer cutting with the cutadapt plugin. Sequences were then quality-filtered, denoised, and merged, followed by the removal of chimera sequences using the DADA2 plugin [15]. Taxonomy was assigned to amplicon sequence variants using the classify-sklearn naïve Bayes taxonomy classifier in the feature-classifier plugin against the Greengenes database [16].

16S rRNA sequencing data were analyzed using the Personalbio GenesCloud (https://www.genescloud.cn/) to conduct bioinformatics analysis. The corrplot package in R language software was used to draw correlation heatmaps. Cytoscape (version 3.9.1) [17] was used to map the correlation network between the top 20 differentially expressed gut microbiota, metabolites, and core proteins related to lipid metabolism, as well as lipid indicators.

### 2.10. Detection of Serum Metabolites in Mice

Mouse serum samples were thawed at 4°C and briefly centrifuged to ensure homogeneity. Aliquots of 100 *μ*L were transferred to 2-mL centrifuge tubes, and 400 *μ*L of methanol (prechilled at −20°C) was added. The mixture was vortexed for 1 min and then centrifuged at 12,000 rpm at 4°C for 10 min. The supernatant was transferred to a new tube, concentrated, and dried. The residue was reconstituted in 150 *μ*L of 80% methanol–water containing 2-chloro-L-phenylalanine (4 ppm) and filtered through a 0.22-*μ*m membrane before LC-MS analysis.

A Thermo Vanquish (Thermo Fisher Scientific, United States) ultrahigh-performance liquid phase system with an ACQUITY UPLC HSST3 (2.1 × 150 mm^2^, 1.8 *μ*m) (Waters, Milford, Massachusetts, United States) column was used, with a flow rate of 0.25 mL/min, a column temperature of 40°C, and an injection volume of 2 *μ*L. In the positive ion model, the Mobile Phases C and D were 0.1% formic acid acetonitrile and 0.1% formic acid water, respectively. The gradient elution procedure was as follows: 0–1 min, 2% mobile phase C; 1–9 min, mobile phase C increased from 2% to 50%; 12–12 min, mobile phase C increased from 50% to 98%; 12–13.5 min, mobile phase C remained at 98%; and 13.5–14 min. In the negative ion mode, the Mobile Phases A and B were acetonitrile and 5 mM ammonium formate water, respectively. The gradient elution procedure was as follows: 0–1 min, 2% mobile phase A; 1–9 min, mobile phase A increased from 2% to 50%; 9–12 min, mobile phase A increased from 50% to 98%; 12–13.5 min, mobile phase A remained at 98%; 13.5–14 min, mobile phase A decreased from 98% to2%; and 14–17 min, mobile phase A remained at 2% A.

Mass spectrometry data were acquired using a Thermo Orbitrap Exploris 120 mass spectrometer (Thermo Fisher Scientific, United States) in both positive and negative ion modes. The positive ion spray voltage was 3.50 kV, and the negative ion spray voltage was −2.50 kV. The sheath gas flow rate was 30 arb, and the auxiliary gas flow rate was 10 arb. The capillary temperature was maintained at 325°C, with a resolution of 60,000 and a scanning range of 100–1000 *m*/*z*. Higher-energy collisional dissociation (HCD) was used for secondary fragmentation with a collision voltage of 30% and a secondary resolution of 15,000. The top four ions were selected for fragmentation, and dynamic exclusion was applied to eliminate redundant MS/MS data.

### 2.11. Data Processing and Analysis of Mouse Serum Metabolomics

Raw mass spectrometry data were converted to mzXML format using the MSConvert tool in the Proteowizard software package (v3.0.8789). Peak detection, filtering, and alignment were performed using the “xcms” package in R [18]. Raw mass spectrometry data were converted to mzXML format using the MSConvert tool in the Proteowizard software package (v3.0.8789). Peak detection, filtering, and alignment were performed using the “xcms” package in R. Parameters were set as follows: bw = 2, ppm = 15, peak width = *c* (5, 30), *m*/*z* width = 0.015, *m*/*z* diff = 0.01, and method = “centWave.” Metabolite identification was conducted using public databases such as the Human Metabolome Database [19], MassBank [20], LipidMaps [21], mzCloud [22], KEGG [23], and a custom-built library, with a mass tolerance of < 30 ppm. Data correction was performed using the LOESS signal [24] correction method based on QC samples, and metabolites with an RSD > 30% in QC samples were excluded. Principal component analysis (PCA), partial least squares–discriminant analysis (PLS-DA), and orthogonal partial least squares–discriminant analysis (OPLS-DA) were conducted using the “Ropls” package [25] in R. Score plots, load plots, and S-plots were generated to visualize metabolite composition differences among samples. Model overfitting was assessed using permutation tests. R2X and R2Y represented the explanatory power of the model for the X and Y matrices, respectively, while Q2 indicated the predictive ability of the model. Metabolites with a *p* value < 0.05 and a VIP value > 1 were considered statistically significant.

Functional pathway enrichment and topological analysis of differential metabolites were performed using the MetaboAnalyst software package [26]. Enriched pathways were visualized using KEGG Mapper to map differential metabolites onto pathway diagrams.

### 2.12. Statistical Analysis

Statistical analyses were conducted using GraphPad Prism software version 5.0. Data are presented as mean ± standard deviation (SD). For univariate analysis, intergroup differences were assessed using one-way analysis of variance (ANOVA), with Tukey's post hoc test applied for multiple comparisons. Comparisons between two groups (e.g., control vs. model group; model vs. YMG group) were performed using the Student *t*-test. A threshold of *p* < 0.05 was used to determine statistical significance.

## 3. Results

### 3.1. Chemical Composition of YMG

**Figure 1:**
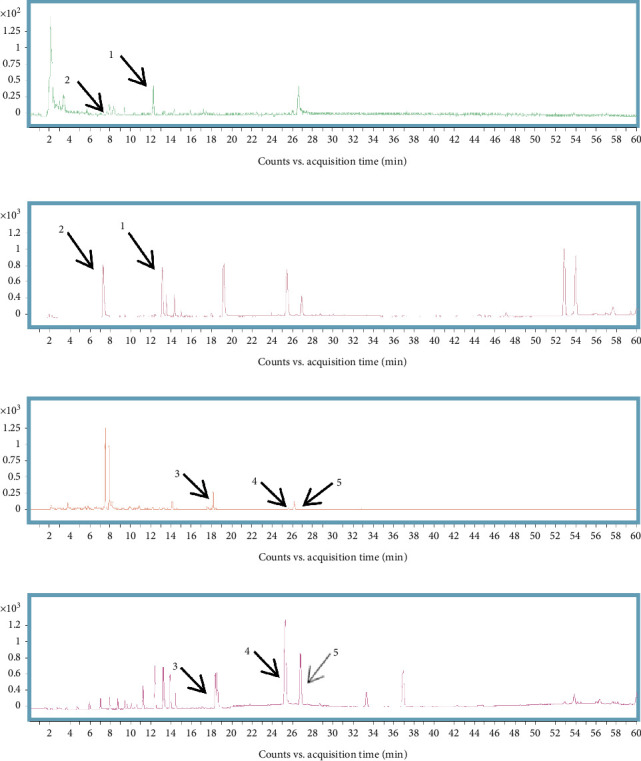
HPLC-ESI-Q-TOF-MS total ion chromatogram of YMG. (a) Total ion chromatogram of YMG in the negative ion mode. (b) Total ion chromatogram of mixed standards in the negative ion mode. (c) Total ion chromatogram of YMG in the positive ion mode. (d) Total ion chromatogram of mixed standards in the positive ion mode. The position of the arrow in the figure is the characteristic peak of the compound.

The HPLC-ESI-Q-TOF-MS total ion chromatogram and results of YMG are shown in Figure 1 and listed in Table 2. A total of five compounds were detected, including three in the positive ion mode and two in the negative ion mode.

### 3.2. Changes in Blood Lipid Levels in Mice Treated With YMG

**Figure 2:**
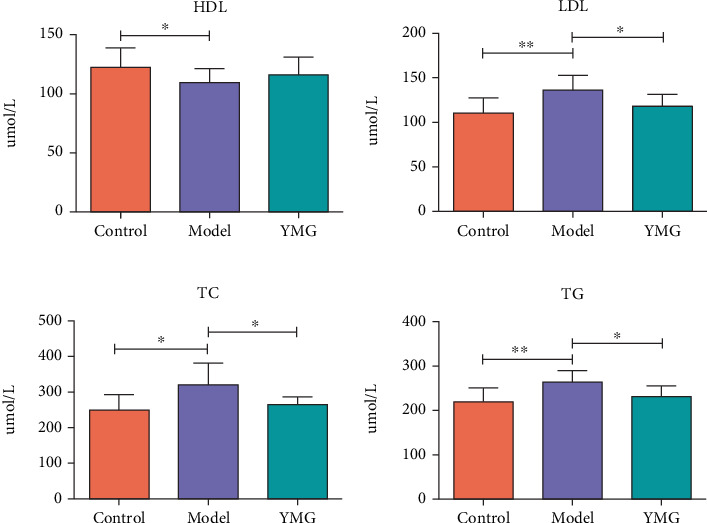
Changes in serum lipid levels in mice treated with YMG. (a) Changes in serum HDL levels in mice. (b) Changes in serum LDL levels in mice. (c) Changes in serum TC levels in mice. (d) Changes in serum TG levels in mice. Data are presented as the mean ± SD (*n* = 6); ⁣^∗^*p* < 0.05 and ⁣^∗∗^*p* < 0.01.

Changes in the serum levels of HDL, LDL, TC, and TG were measured to evaluate the improvement in blood lipid levels in mice treated with YMG. As shown in Figure 2, the LDL, TG, and TC levels in the model group significantly increased compared to those in the control group (*p* < 0.01 for LDL and TG and *p* < 0.05 for TC). The difference in HDL levels between the model and control groups was not significant (*p* > 0.05). The LDL, TC, and TG levels in the YMG group decreased significantly compared to those in the model group (*p* < 0.05), whereas HDL levels were not significantly different (*p* > 0.05).

### 3.3. Pathological Observation of Mouse Liver

**Figure 3:**
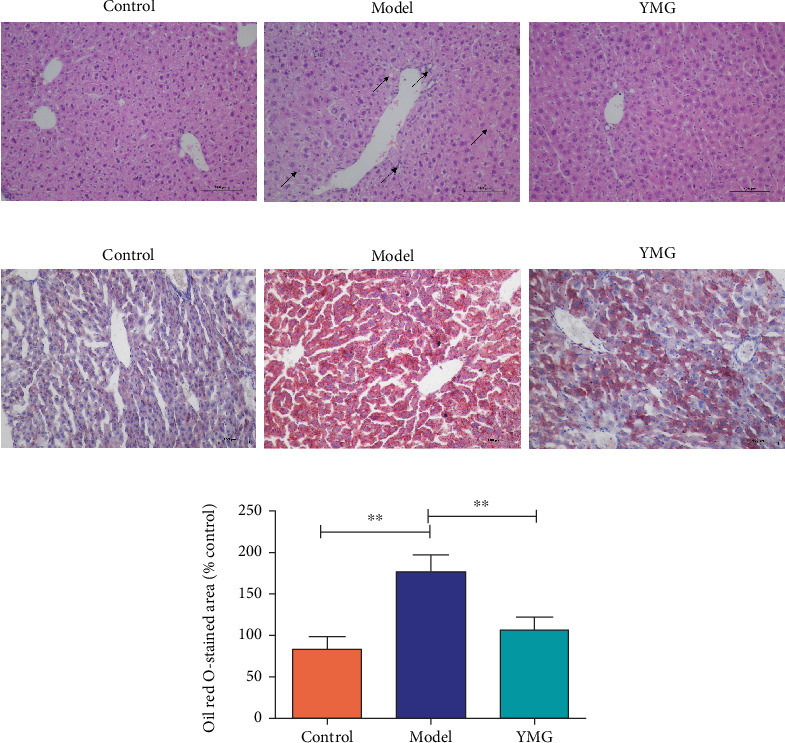
(a) Hematoxylin and eosin staining of mouse liver tissues with different treatments (*n* = 6; × 200 magnification). (b) Oil red O-stained liver sections (*n* = 6; × 400 magnification). (c) Semiquantification of the Oil Red O–stained area. Data are presented as the mean ± SD (*n* = 6); ⁣^∗^*p* < 0.05 and ⁣^∗∗^*p* < 0.01.

As shown in Figure 3a, the mouse liver cells in the control group were arranged densely and uniformly with normal structure, whereas those of the model group exhibited lighter staining, different nuclear sizes, hepatocyte edema, necrosis, and fragmentation of some cells, as well as a small amount of fat vacuole formation. In contrast, the degree of lesions in the YMG group decreased significantly, with weakened hepatocyte enlargement, fewer fat vacuoles, and the reversal of changes in hepatic sinuses, compared to that in the model group.

Oil Red O staining was used to demonstrate the deposition of liver fat in mice (Figure 3b). The relative lipid area (percentage) in the model group was significantly increased (*p* < 0.05) (Figure 3c) compared to that in the control group, and the relative lipid area (percentage) in the YMG group was significantly decreased (*p* < 0.05) (Figure 3c).

### 3.4. YMG Network Pharmacological Analysis

**Figure 4:**
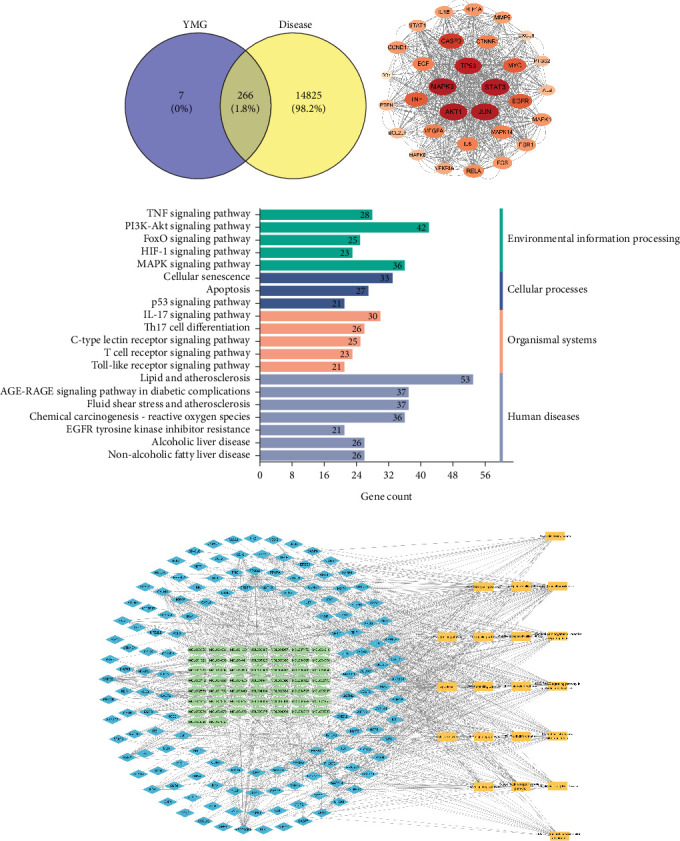
(a) Venn diagram of YMG and disease intersecting targets. (b) PPI network diagram of core targets. Red is the core target. (c) KEGG pathway enrichment analysis. The horizontal axis is the number of core targets on the pathway, and the vertical axis is the pathway in KEGG. Different colors indicate the classification of different functions of KEGG pathway. (d) Network analysis diagram of the compound–target–signal pathway. Yellow is the metabolic pathway, blue is the core target, and green is the compound of traditional Chinese medicine.

After screening in the TCMSP database, a total of 224 effective compounds in YMG were obtained, including 13 species of *Pinellia ternata*, 21 species of *Codonopsis pilosula*, 20 species of *Radix Astragali*, 15 species of *Poria cocos*, seven species of *Atractylodes macrocephala*, 92 species of *licorice*, 11 species of *Trichosanthes*, 23 species of peach kernel, and 22 species of safflower. After deleting the duplicates, 203 effective compounds of YMG and 279 corresponding targets remained. A total of 1682; 15,050; and 471 dyslipidemia targets were obtained from the GeneCards, CTD, and DisGeNET databases, respectively, and 15,091 disease targets were obtained after weight deletion. A total of 266 YMG targets intersected (Figure 4a).

The intersecting targets were imported into the STRING database to obtain a PPI network, which was visualized using the CytoHubba plugin. The top 40 MNC, Degree, EPC, and MCC targets were selected and intersected, obtaining 31 core targets (Table 3), which were then imported into the STRING database to obtain a PPI network for the core targets. The PPI network included 31 nodes and 652 edges (Figure 4b).

KEGG pathway enrichment analysis showed 182 signaling pathways, including the PI3K-Akt, TNF, IL17, apoptosis, NAFLD, and lipid and atherosclerosis signaling pathways (Figure 4c).

A compound–target–pathway network was constructed using the top 20 signaling pathways and their corresponding targets and compounds, as shown in Figure 4d, where the green circles, blue diamonds, and orange rectangles represent the compounds, targets, and pathways, respectively. Based on Table 3 and compound–target–signal pathway analysis, Western blot validation was performed for caspase-3, CYCS, IL1*β*, IL6, PPAR, RXRA, TNF, and NF-*κ*B 65 proteins related to NAFLD in the screening pathways.

### 3.5. Western Blot Detection of Core Protein Expression

**Figure 5:**
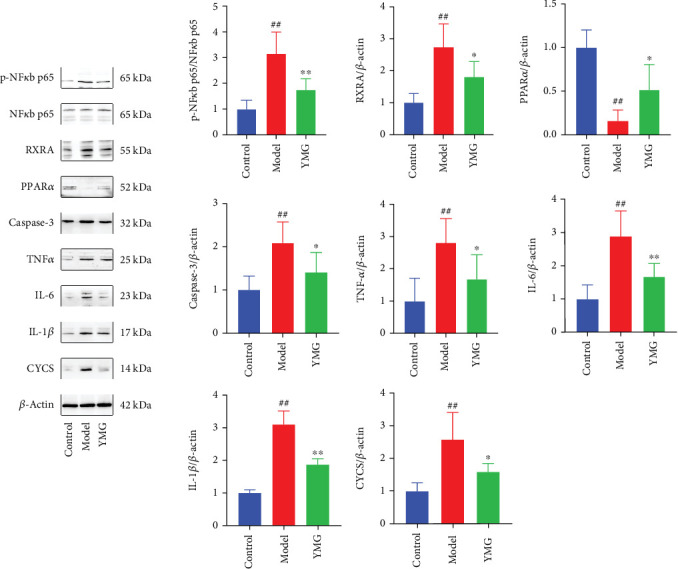
Western blot detection of core protein expression. Data are presented as the mean ± SD (*n* = 6); ⁣^∗^*p* < 0.05 and ⁣^∗∗^*p* < 0.01 compared to the control group; ^##^*p* < 0.01 compared to the model group.

As shown in Figure 5, based on Table 3 and compound–target–signal pathway analysis, Western blot validation was performed on caspase-3, CYCS, IL1*β*, IL6, PPAR*α*, RXRA, TNF, and NF-*κ*B 65 proteins related to NAFLD in the screening pathways. The model group showed a significant increase in the protein expression levels of p-NF-*κβ* 65/NF-*κβ* 65, RXRA, caspase-3, TNF, CYCS, IL1 *β*, and IL6 compared to the control group (*p* < 0.01). The YMG group exhibited a significant reduction in p-NF-*κβ* 65/NF-*κβ* 65, IL6, and IL1*β* protein expression levels (*p* < 0.01), along with reduced expressions of RXRA, TNF, and CYCS proteins (*p* < 0.05) and a significant increase in PPAR*α* protein expression (*p* < 0.01).

### 3.6. Effects of YMG on the Gut Microbiota of Mice

**Figure 6:**
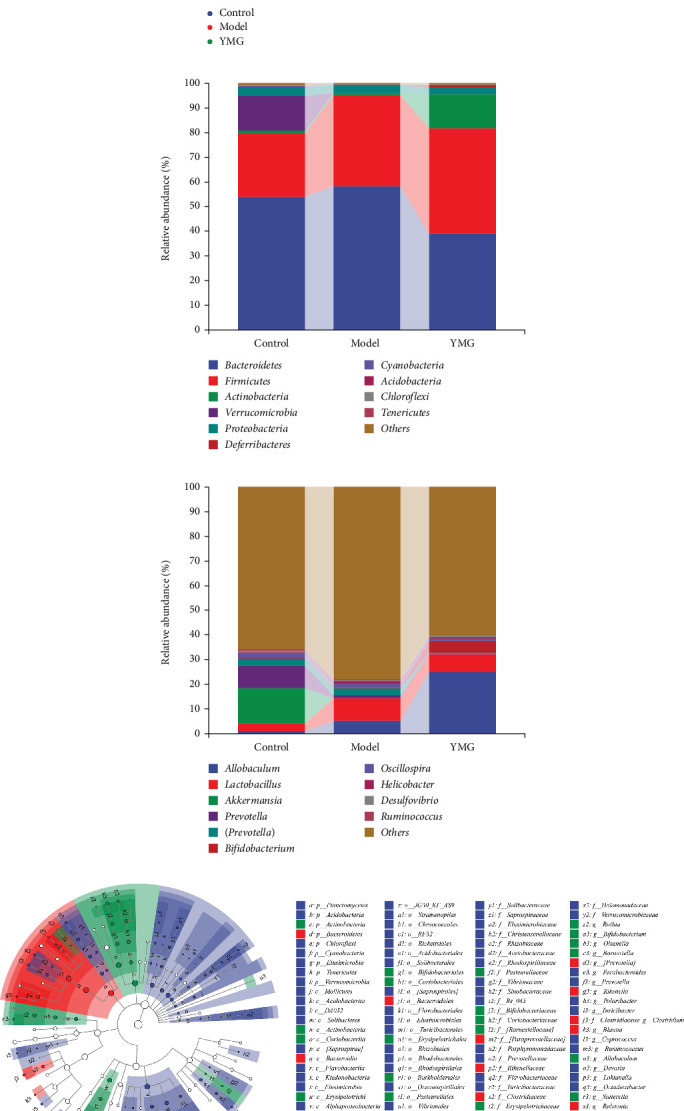
Effects of YMG on the gut microbiota in mice; *n* = 6. (a) Alpha-diversity analysis of gut microbiota. The Kruskal–Wallis rank sum test and Dunnett's test were used as post hoc tests to verify the significance of the difference.∗*p* < 0.05 and ⁣^∗∗^*p* < 0.01. (b) Beta-diversity PCoA of gut microbiota. PCoA based on Bray–Curtis distance. The ellipse confidence was 0.95. (c) Abundance of the top 10 bacterial phyla in mice. (d) Abundance of the top 10 bacterial genera in mice. (e) Analysis of differences in the gut microbiota based on the LEfSe cladogram. Linear discriminant analysis score threshold ≥ 2. (f) Predictive analysis of gut microbial function using PICRUSt2. Data are presented as the mean ± SD (*n* = 6); *p* value of the overall difference between groups obtained by the Kruskal–Wallis nonparametric test. ⁣^∗^*p* < 0.05 and ⁣^∗∗^*p* < 0.01.

The changes in the gut microbiota of mice after YMG treatment were evaluated using *16S rRNA* gene sequencing. YMG significantly reduced the Chao1 and Shannon indices of the mouse gut microbiota compared to the control group, and the Simpson index of the model group was significantly higher. This indicated significant changes in both the richness and evenness of gut microbiota among the different subgroups (Figure 6a).

PCoA based on the Bray–Curtis distance showed obvious differences in the mouse gut microbiota among different groups (Figure 6b). At the same time, the Adonis results of *β*-diversity showed *p* < 0.001. This indicated that the different treatments significantly altered the composition of the gut microbiota in the different groups.

The annotated results of gut microbiota at the phylum and genus levels are shown in Figure 6c,d. The dominant species in the mouse gut microbiota at the phylum level included Bacteroidetes, Firmicutes, Actinobacteria, Verrucomimicrobia, and Proteobacteria, while those at the genus level included *Allobaculum*, *Lactobacillus*, *Akkermansia*, *Prevotella*, *Bifidobacterium*, *Oscillospira*, *Helicobacter*, *Desulfovibrio*, and *Ruminococcus*. The gut microbiota at the phylum and genus levels showed significant changes among the groups.

Linear discriminant analysis effect size (LEfSe) analysis was performed to determine the significance of differences in gut microbiota at different classification levels (Figure 6). The LEfSe analysis showed nine and 22 differential microorganisms at the phylum and genus levels, respectively. At the genus level, the differential gut microbiota closely related to NAFLD were *Lactobacillus*, *Akkermansia*, *Bifidobacterium*, *Prevotella*, *Ruminococcus*, *Bacteroides*, *Parabacteroides*, *Blautia*, and *Bilophila*.

A functional prediction of gut microbiota was conducted using PICRUSt2 to further determine whether significant changes in the gut microbiota in the treatment groups altered microbial function. The PICRUSt2 functional prediction results showed significant differences in lipid and carbohydrate metabolism among the different treatment groups (*p* < 0.05). This indicated that the gut microbiota in the different treatment groups significantly affected lipid and carbohydrate metabolic functions.

### 3.7. Effects of YMG on Blood Metabolites in Mice

**Figure 7:**
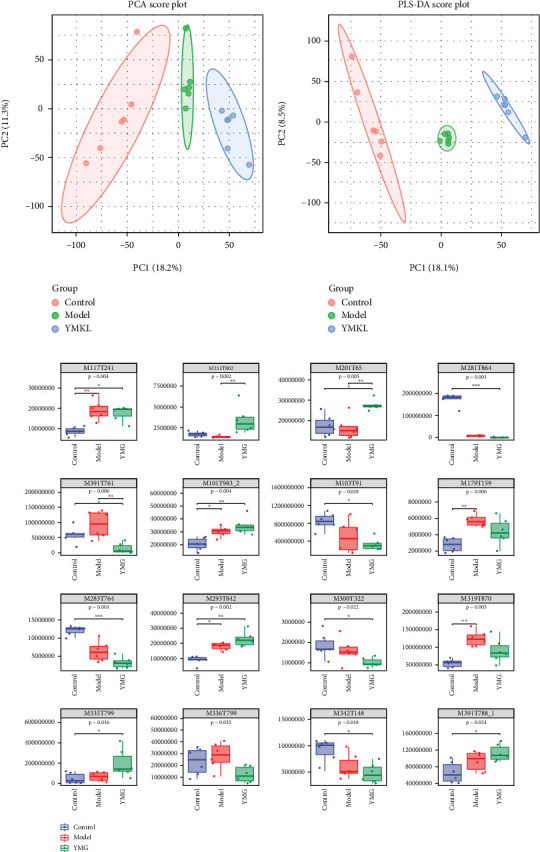
Effects of YMG on serum metabolites in mice; *n* = 6. (a) PCA of mouse serum metabolites. The ellipse confidence was 0.95. (b) PLS-DA analysis of mouse serum metabolites. The ellipse confidence was 0.95. (c) Differential analysis of metabolites in mouse serum. The Kruskal–Wallis rank sum test and Dunnett's test were used as post hoc tests to verify the significance of the difference. ⁣^∗^*p* < 0.05, ⁣^∗∗^*p* < 0.01, and ⁣^∗∗∗^*p* < 0.001.

A total of 3321 and 3131 metabolites were detected in the positive and negative ion modes, respectively. PCA (Figure 7a) and PLS-DA (Figure 7b) showed obvious clustering among the different treatment groups. This indicated significant differences in the metabolite composition in the different treatment groups. A total of 92 metabolites were significantly different among the groups. KEGG annotation suggested that 14 metabolites were related to lipid and carbohydrate metabolism (Table 4).  Figure 7c shows the changes in these 14 differential metabolites.

### 3.8. Construction of a Microbial Metabolite Lipid Regulatory Network to Improve NAFLD Using YMG

**Figure 8:**
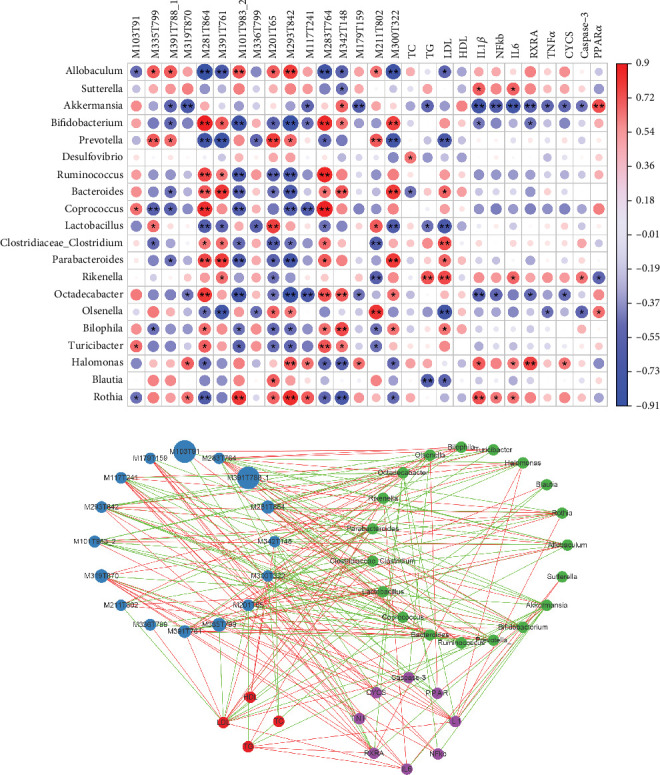
Correlation analysis of the top 20 differential gut microbiota and metabolites, blood lipid indicators, and core proteins; *n* = 6. (a) Correlation heat map analysis of the top 20 differential gut microbiota, differential blood metabolites, blood lipid indicators, and core proteins; ⁣^∗^*p* < 0.05 and ⁣^∗∗^*p* < 0.01. (b) Correlation network analysis of the correlation between the top 20 differential gut microbiota, differential metabolites, blood lipid indicators, and core proteins. The red and green lines indicate positive and negative correlations, respectively. Nodes showed *p* < 0.05 and *R* > 0.5.

Spearman's correlation heat map was constructed, and correlation network analysis was performed to explore the mechanism of YMG in regulating the gut microbiota and metabolites on improving NAFLD. The heat map (Figure 8a) showed a correlation between the blood lipid indicators and the top 20 differential gut microbiota, metabolites, and core proteins. The correlation network analysis showed differences among blood lipid indicators, the gut microbiota, and metabolites. This indicated that the top 20 differential gut microbiota had different regulatory effects on metabolites, core proteins, and blood lipid indicators.

An association network was plotted based on Spearman analysis to further explore the complex regulatory relationship among the gut microbiota, differential metabolites, core proteins, and blood lipid indicators (*p* < 0.05, *R* > 0.5) (Figure 8b). The association network showed a correlation between blood lipid indicators in mice, core proteins, the top 20 differential gut microbiota, and metabolites, including both positive and negative correlations. The results indicated that YMG might have a regulatory effect on blood lipids in NAFLD mice through various gut microbiota and metabolites.

## 4. Discussion

The gut microbiota plays a key role in regulating host physiological metabolism, as well as the occurrence and development of various diseases [27]. Numerous gut microbiota are potential targets of TCM for disease intervention and treatment [11, 28, 29]. Clarifying the mechanism of action of TCMs and their active ingredients is challenging due to the synergistic effects of the complex components in TCM on multiple targets of disease. Therefore, network pharmacology and various combined sequencing means were used to screen the potential targets of TCMs for disease intervention and treatment to clarify their complex mechanisms. In this study, the dosage of YMG was derived by converting the ratio of human body surface area to animal body surface area. We did not set different concentrations for the dosage of YMG. This is primarily because YMG has already been applied to clinical patients, and we are more concerned about the impact of the clinical dosage of YMG on NAFLD. Additionally, due to the complex composition of YMG, its molecular mechanism differs from that of conventional drugs used for the clinical treatment of NAFLD. We primarily focus on exploring the mechanism of YMG in treating NAFLD. Therefore, positive control drugs were also not included in this study. YMG significantly improved the clinical symptoms of NAFLD, which was observed in liver tissue pathological sections and related blood lipid indices. Network pharmacology showed that the effect of YMG to improve NAFLD might be due to numerous chemical compounds. KEGG analysis in network pharmacology showed that these compounds were enriched in the NAFLD pathway, as well as other pathways related to inflammation and apoptosis. Western blots confirmed the accuracy of the target proteins in these pathways. These results suggested that YMG might exert other effects on NAFLD, which should be explored in future studies. YMG could also regulate the gut microbiota and blood metabolites involved in lipid metabolism in NAFLD mice. Correlation heat map and association network analyses showed that some key gut microbiota were not strongly correlated with HDL levels. This result is consistent with the results of our blood lipid testing, in which HDL levels did not differ significantly between the three groups.

The current study indicated that during the development of NAFLD, YMG improved gut microbial diversity and reduced some alpha-diversity indices. Generally, an abundant gut microbiota is linked to robust health. Nonetheless, species variations may yield distinct outcomes and disease presentations often associated with some strains [30]. Consequently, exploring variations in specific strains under each genus is imperative for future investigation. The abundance of *Lactobacillus*, *Akkermansia*, *Bifidobacterium*, *Prevotella*, *Ruminococcus*, *Bacteroides*, *Parabacteroides*, *Blautia*, and *Bilophila* notably changed at the genus level in NAFLD mice. *Akkermansia*, a prevalent probiotic under study, is potentially suited for metabolic syndrome therapy. It improves metabolic dysregulation in obese animals and humans by reducing insulin sensitivity and dyslipidemia [31, 32]. The abundance of *Akkermansia* decreased in the model group compared with the control group, and we suspect that *Akkermansia* may have a potential role in the development of NAFLD disease. However, YMG did not increase the abundance of *Akkermansia*. Therefore, further research is needed to determine whether *Akkermansia* is a target of YMG treatment. Some studies have shown that the occurrence and development of NAFLD were closely related to specific changes in the gut microbiota, including Bacillota and Bacteroidota [33, 34], while some adverse diets caused gastrointestinal mucosal inflammation and damage to the intestinal mucosal barrier, increasing the probability of translocating bacteria and their products, thereby promoting the occurrence of NAFLD [35]. In addition, gut microbial metabolites also affect the development of NAFLD, indicating their crucial role in NAFLD [3, 36, 37]. *16S rRNA* gene sequencing analysis showed that the gut microbiota and their function changed significantly in *ApoE*^−/−^ mice after the administration of YMG. Significant changes were also observed in the blood lipid indicators and pathological section results of *ApoE*^−/−^ mice. These results suggest that YMG could improve HFD-induced NAFLD through the gut microbiota. The correlation heat maps and correlation networks also confirmed these results. Among the differential gut microbiota, *Lactobacillus*, *Akkermansia*, *Bifidobacterium*, *Prevotella*, *Ruminococcus*, *Bacteroides*, *Parabacteroides*, *Blautia*, and *Bilophila* were reported to be closely related to obesity and NAFLD [6, 29]. Obese and NAFLD patients exhibited an increased abundance of *Prevotella*. However, views on the relationship between *Prevotella* and diseases differ. Some studies showed that an increase in *Prevotella* abundance could promote glycogen storage and produce succinic acid, activating gluconeogenesis in the intestine, which is closely related to sugar metabolism and insulin tolerance [38, 39]. However, other studies found that *Prevotella* could regulate the biosynthesis of branched-chain amino acids, leading to the occurrence and development of Type 2 diabetes [40]. In addition, an increase in *Prevotella* abundance induced stromal cells to release more inflammatory mediators, thereby promoting the occurrence of chronic inflammation [41]. After the administration of YMG, the intestinal abundance of *Prevotella* significantly increased, and the correlation heat map showed a significant negative correlation between *Prevotella* abundance and LDL levels, indicating that YMG might play a certain LDL regulatory role through *Prevotella*. Similar to *Prevotella*, *Blautia* is also a controversial microorganism. It has anti-inflammatory effects and helps repair intestinal mucosal damage [42, 43]. An increase in *Blautia* abundance was reported to reduce visceral fat content, playing a certain therapeutic role in some metabolic disorders [44]. However, some studies have shown that *Blautia* abundance is positively related to intestinal permeability, as well as some diagnostic markers of cardiovascular disease, such as plasma glutamic acid and branched-chain amino acid [45, 46]. *Blautia* abundance was reported to increase in diseases like obesity and NASH [47, 48]. However, in the current study, the *Blautia* abundance in NAFLD mice increased and further increased after the administration of YMG to the mice. These results might be due to the inability to achieve finer *Blautia* resolution using *16S rRNA* gene sequencing in the current study. *Parabacteroides* are short-chain fatty acid–producing microorganisms that have anti-inflammatory properties [49]. The abundance of *Parabacteroides* was increased in patients with obesity and NAFLD [49]. *Ruminococcus* increases intestinal energy absorption by regulating resistant starch, thereby promoting weight gain [50]. *Ruminococcus* can also induce oxidative stress and inflammatory reactions, affecting human intestinal health [51, 52]. In the current study, significant changes were observed in the abundance of *Ruminococcus*. However, the results of the correlation heat map showed that *Ruminococcus* did not have a strong correlation with blood lipid-related indicators. However, considering the interaction between gut microbiota and other NAFLD disease phenotypes, *Ruminococcus* requires further investigation. The current study showed that the changes in the gut microbiota of NAFLD model mice before and after the administration of YMG were not consistent with the results of numerous other studies, which might be related to disease status, animal models, administration methods, and the feeding environment; the influence of gut microbiota on the body is bidirectional. Due to the complexity of the gut microbiota, only a few disease phenotypes were associated with a few strains, and the impact of gut microbiota on diseases may result from the joint action of multiple microorganisms. Therefore, the mechanism of disease regulation after administering YMG to NAFLD model mice, in combination with other experiments, requires further investigation.

Metabolomics can detect small molecule compounds in the body, which can serve as targets for disease diagnosis. In addition, these small molecule compounds also play a crucial role in the occurrence and development of obesity and NAFLD. A study by Li et al. found that non-12*α*-hydroxylated produced by *Parabacteroides distasonis* was closely related to the weight gain of mice after caloric restriction [53]. The weight rebound of mice could be effectively inhibited by feeding *Parabacteroides distasonis* or non-12*α*-hydroxylated. Another study showed that betaine supplementation to HFD-induced obese mice reduced the disorder of gut microbiota and improved obesity [54]. The gut microbial metabolites lipopolysaccharide, small-chain fatty acids, bile acid, and tryptophan are also key substances regulating the occurrence and development of NAFLD [9, 36, 55, 56]. In the current study, nontargeted metabolomics were used to detect metabolites in mouse blood. A total of 14 metabolites related to lipid and carbohydrate metabolism were screened through the annotation of KEGG metabolites. These metabolites might be closely related to the metabolism of blood lipids. Among them, some metabolites play a crucial role in the pathogenesis of dyslipidemia and hyperlipidemia. Studies have shown that linoleic acid can reduce serum cholesterol levels and slight structural changes in early liver tissue in hypercholesterolemic rats [57]. Similarly, research has found that trehalose can reshape the gut microbiota to regulate hyperlipidemia [58]. Meanwhile, allocholic acid has been found to effectively reduce aspartate aminotransferase levels and regulate fat digestion, cholesterol metabolism, liver function, and hepatobiliary circulation [59]. In our study, we found a significant negative correlation between dodecanoic acid and HDL and TG, which is similar to previous research findings. It has been found that dodecanoic acid is closely related to impaired liver function and inflammation and may be a novel biomarker for NAFLD [60]. Furthermore, research on D-mannose has also found that it can combat NAFLD by reducing the expression of genes regulating liver fat production [61]. Although the remaining metabolites in Table 4 have not been reported to be related to NAFLD, our results indicate their relationships with microorganisms, core targets, and lipid indicators. We will further focus on these in our subsequent research. The blood lipid indicators HDL, LDL, TC, and TG can be used as standards for evaluating NAFLD. The potential prediction of functional gut microbiota showed that the lipid and carbohydrate metabolisms of the gut microbiota changed significantly after YMG administration. The correlation heat map and network analyses showed a complex regulatory relationship among differential gut microbiota, metabolites, and blood lipid indicators. Blood lipid indicators might be jointly regulated by the gut microbiota and metabolites. The correlation heatmaps and correlation network graphs also indicated a complex relationship between the gut microbiota, metabolites, lipid indicators, and network pharmacology core proteins. YMG might have a regulatory effect on this complex relationship, which requires further research.

This study had certain limitations. The effects of the active ingredients screened by YMG network pharmacology in NAFLD model mice were not elucidated, and these will be further studied in subsequent experiments. More precise identification of the gut microbiota and clear regulatory relationships among the gut microbiota, metabolites, and blood lipid indicators also need to be verified through subsequent experiments, such as metagenomics, targeted metabolomics, fecal microbial transplantation, and antibiotic intervention. However, the current study elucidated the complex regulatory mechanism of the multiple active ingredients in YMG in improving NAFLD through the gut microbiota and metabolites.

## 5. Conclusions

In summary, this study demonstrated that YMG could improve the pathology of HFD-induced NAFLD mice and showed that these regulatory effects were related to changes in gut microbial function and serum metabolites. Meanwhile, network pharmacology results indicate that YMG has a certain regulatory effect on the nonalcoholic fatty liver disease pathway and its core targets. However, due to the complexity of TMC ingredients and regulatory mechanisms, the molecular mechanism of YMG in improving NAFLD requires further elucidation. The current study clearly highlighted the therapeutic value of YMG for ameliorating NAFLD progression. In addition, this study provided a new idea for studying the mechanism of TCM prescriptions in the treatment of NAFLD using bioinformatics methods. This study might provide a theoretical basis for the clinical application of YMG in the treatment of NAFLD.

## Figures and Tables

**Table 1:** Composition of TCM in YMG.

**Traditional Chinese medicine**	**Latin name**	**Weight**
DangSen	*Codonopsis pilosula* (Franch.) Nannf.	10 g
Baizhu	*Atractylodes macrocephala* Koidz.	10 g
FuLin	*Poria*	10 g
Gancao	*Radix Glycyrrhizae*	3 g
HuangQi	*Astragali Radix*	10 g
TaoRen	*Semen Persicae*	10 g
HongHua	*Carthamus tinctorius* L.	6 g
QingBanxia	*Pinelliae Rhizoma Praeparatum Cum Alumine*	6 g
Gualou	*Trichosanthis Fructus*	10 g

**Table 2:** HPLC-ESI-Q-TOF-MS results of the qualitative analysis of YMG.

**Number**	**Chemical compound**	**Chemical formula**	**Peak standard curve area**	**Standard curve concentration (mg/mL)**	**Content (mg/g)**
1	*trans*-ferulic acid	C_10_H_10_O4	37762236	0.46	0.0941
2	Chlorogenic acid	C_16_H_18_O_9_	15307857	0.42	0.8163
3	Calycosin-7-O-*β*-D-glucoside	C_22_H_22_O10	89027132	0.48	0.9376
4	Formononetin	C16H12O4	10874517	0.5	5.5526
5	(3R)-8,2⁣′-Dihydroxy-7,4⁣′-dimethoxyisoflavan	C17H18O5	59419133	0.5	0.0995

**Table 3:** Topological analysis of core targets.

**No.**	**UniProt ID**	**Gene name**	**MCC/× 10** ^**7**^	**MNC**	**Degree**	**EPC**
1	P40763	STAT3	336.00	68	136	27.512
2	P15692	VEGFA	238.86	45	90	21.385
3	P27361	MAPK3	215.43	62	124	24.638
4	P04637	TP53	213.99	75	150	27.202
5	P31749	AKT1	213.20	76	152	27.436
6	P05412	JUN	212.12	69	138	27.274
7	P42574	CASP3	184.18	55	110	23.798
8	P35222	CTNNB1	177.91	52	106	22.958
9	P24385	CCND1	176.04	43	86	21.446
10	P01106	MYC	175.62	49	100	23.74
11	P60484	PTEN	154.45	34	68	18.511
12	P00533	EGFR	152.91	55	112	23.804
13	P01375	TNF	152.02	62	124	25.12
14	P05231	IL6	152.00	53	106	23.603
15	P01584	IL1*β*	124.90	45	92	20.26
16	P01133	EGF	120.60	37	74	20.155
17	P10145	CXCL8	120.46	32	64	16.554
18	Q16665	HIF1A	71.94	39	80	21.061
19	Q07817	BCL2L1	49.17	33	68	17.66
20	P14780	MMP9	38.04	40	80	18.712
21	P03372	ESR1	27.83	46	92	21.523
22	P02768	ALB	23.54	40	84	18.336
23	P01100	FOS	21.87	42	84	21.321
24	P01100	RELA	18.68	50	100	23.532
25	Q04206	PTGS2	15.61	28	66	18.459
26	P35354	MAPK14	13.88	38	76	20.119
27	Q16539	STAT1	8.83	35	70	19.806
28	P42224	MAPK1	8.15	51	102	22.485
29	P28482	NFKBIA	3.77	33	66	18.761
30	P25963	MAPK8	2.74	41	82	20.457
31	P45983	RB1	1.70	33	66	16.947

**Table 4:** Differential metabolites in mouse serum.

**ID**	**Name**	**KEGG**	**KEGG_pathway**
M117T241	Deoxyribose	C01801	Carbohydrate metabolism
M211T802	(-)-Jasmonic acid	C08491	Lipid metabolism
M201T65	Dodecanoic acid	C02679	Lipid metabolism
M281T864	Linoleic acid	C01595	Lipid metabolism
M391T761	Allocholic acid	C00695	Lipid metabolism
M101T983_2	Succinic acid semialdehyde	C00232	Carbohydrate metabolism
M103T91	(R)-3-Hydroxybutyric acid	C01089	Carbohydrate metabolism
M179T159	D-Mannose	C00159	Carbohydrate metabolism
M283T764	Stearic acid	C01530	Lipid metabolism
M293T842	13-L-Hydroperoxylinoleic acid	C04717	Lipid metabolism
M300T322	2-Methoxyestrone	C05299	Lipid metabolism
M319T870	20-HETE	C14748	Lipid metabolism
M335T799	Hepoxilin B3	C14810	Lipid metabolism
M336T799	12(R)-HPETE	C14812	Lipid metabolism
M342T148	Trehalose	C01083	Carbohydrate metabolism
M391T788_1	Chenodeoxycholic acid	C02528	Lipid metabolism

## Data Availability

The data used to support the findings of this study are available from the corresponding author upon request.
